# Towards Wearable-Inertial-Sensor-Based Gait Posture Evaluation for Subjects with Unbalanced Gaits

**DOI:** 10.3390/s20041193

**Published:** 2020-02-21

**Authors:** Sen Qiu, Huihui Wang, Jie Li, Hongyu Zhao, Zhelong Wang, Jiaxin Wang, Qiong Wang, Dirk Plettemeier, Michael Bärhold, Tony Bauer, Bo Ru

**Affiliations:** 1 Key Laboratory of Intelligent Control and Optimization for Industrial Equipment of Ministry of Education, Dalian University of Technology, Dalian 116024, China; 1165530693@mail.dlut.edu.cn (J.L.); zhaohy@dlut.edu.cn (H.Z.); wangzl@dlut.edu.cn (Z.W.); wangjx19890828@mail.dlut.edu.cn (J.W.); 2 School of Control Science and Engineering, Dalian University of Technology, Dalian 116024, China; natalie@mail.dlut.edu.cn; 3 School of Fundamental Education, Dalian Neusoft University of Information, Dalian 116023, China; 4 Chair for Radio Frequency and Photonics Engineering, Communication Laboratory, Technische Universität Dresden, 01062 Dresden, Germany; qiong.wang@tu-dresden.de (Q.W.); dirk.plettemeier@tu-dresden.de (D.P.); michael.baerhold@tu-dresden.de (M.B.); tony.bauer@tu-dresden.de (T.B.)

**Keywords:** MEMS sensors, information fusion, gait analysis, rehabilitation assessment, body sensor network

## Abstract

Human gait reflects health condition and is widely adopted as a diagnostic basis in clinical practice. This research adopts compact inertial sensor nodes to monitor the function of human lower limbs, which implies the most fundamental locomotion ability. The proposed wearable gait analysis system captures limb motion and reconstructs 3D models with high accuracy. It can output the kinematic parameters of joint flexion and extension, as well as the displacement data of human limbs. The experimental results provide strong support for quick access to accurate human gait data. This paper aims to provide a clue for how to learn more about gait posture and how wearable gait analysis can enhance clinical outcomes. With an ever-expanding gait database, it is possible to help physiotherapists to quickly discover the causes of abnormal gaits, sports injury risks, and chronic pain, and provides guidance for arranging personalized rehabilitation programs for patients. The proposed framework may eventually become a useful tool for continually monitoring spatio-temporal gait parameters and decision-making in an ambulatory environment.

## 1. Introduction

Human motion monitoring has become a research hotspot as the world population has aged. The elderly normally suffer from loss of mobility due to muscle weakness or body joint disorders, which results in high risk of falling or other physical consequences. The above-mentioned situation is dangerous, especially for the older adults who live alone. Ambulatory monitoring of human limb motion offers an effective and low-cost solution to early diagnosis of human limb disorders via wireless wearable sensors and pervasive computing [1,2,3,4].

**Figure 1:**
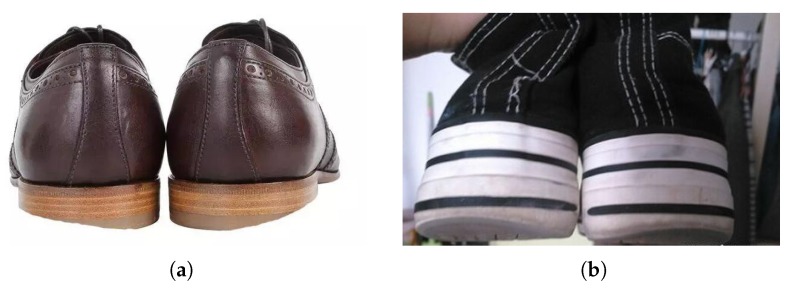
Abnormal gaits reflected in the soles of shoes. (**a**) Strephenopodia is present on both sides; (**b**) apparent asymmetry on both sides.

Walking is one of the most basic means of human locomotion, and is often overlooked by most individuals. Actually, the way of walking or jogging can affect a person’s life significantly. The walking manner, i.e., the human gait, has a big influence on our capacity for independent living and on health issues in the long term. Acknowledging the importance of gait analysis is merely the first step; then, we need to understand the essentials of gait analysis. Many people take it for granted that gait analysis is just about someone observing you walk or run and evaluating your shoes and your feet. Consider a purchasing scenario in which you are going to buy shoes in a store; a clerk checks the shoes on your feet, as shown in Figure 1, and suggests a pair of shoes that are most suitable for you. However, this is far from revealing the biomechanical mechanism. Gait is the characteristic of human walking. Clinical gait analysis is designed to reveal the key links and influencing factors of gait anomalies through biomechanics and kinematics, to help evaluate and treat through rehabilitation, and to help to assist clinical diagnosis, curative effect evaluation, and pathological research, etc. [5,6].

An effective gait analysis is usually performed in clinical or research settings and quantitatively measures the exact angles of the knees, ankles, and hips when walking or running. Note that traditional gait analysis performed in a specific gait lab is too expensive and inconvenient. It is necessary to develop an ambulatory gait analysis method without a complex process, which is helpful for physiotherapists to easily discover abnormal gaits and the causes of chronic gait pain, as well as for fitness instructors to discover exercise injury risks and to provide guidance [7,8,9].

## 2. Related Works

**Figure 2:**
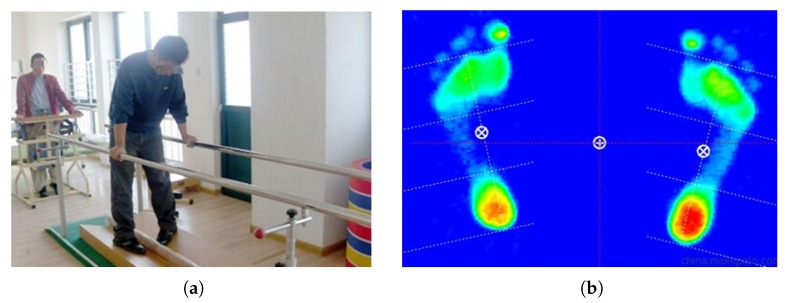
Traditional gait analysis methods: (**a**) Physician observation; (**b**) plantar pressure monitoring.

Traditional gait analysis is based on the therapist’s observation and judgment, as shown in Figure 2a. This method relies heavily on the therapist’s experience; the assessment may be inaccurate, partly because these behavioral and clinical assessments are subjective [10]. In addition, the plantar pressure monitoring method offers details of plantar pressure; however, it cannot record the gait parameters in the swing phase (such as the foot speed, walking trajectory, joint angle, etc.), which is not conducive to the determination and improvement of the lower-limb treatment plan [11,12,13].

At present, the precision of multiple-camera-based gait analysis is widely acknowledged, such as with the commercial products Vicon and Optitrack. However, the cost of a complete set of multiple-camera systems is high. The marking points should be worn in specific positions on the human body according to the principles of human anatomy. Although optical gait analysis technology has achieved the highest-recognized accuracy, it must rely on the existence of indoor infrastructure, and the operation process is normally time consuming and labor intensive [14,15]. In addition, the laboratory environment does not reflect patients’ real living conditions and mobility needs. It is obvious that laboratory-level gait analysis can only analyze a very limited number of steps on horizontal ground. As a result, gait analysis in the laboratory is often limited. All the above factors indicate the need for a new gait analysis method with real clinical value [16,17].

With the rapid development of micro-electro-mechanical systems (MEMS), the functions and performance of wearable inertial sensors have become increasingly powerful. As low-cost and compact sensors with low power consumption have been manufactured, inertial measurement units (IMUs) consisting of accelerometers, gyroscopes, and magnetometers have become more accurate and easier to implement. Novel gait analysis methods estimate gait parameters on the basis of the kinematic characteristics of subjects’ lower limbs to estimate the step length, joint angle symmetry, and balance at each step [18]. Wearable gait analysis technology does not rely on any external infrastructure and additional environmental information, which is a cost-effective choice and provides a new direction for gait related research [19]. Portable gait analysis systems use miniature inertial sensors and wireless communication systems to efficiently capture the motions of patients’ lower limbs. Human motion capture systems based on IMU are not affected by light, space, or other restrictions. An inertial pediatric smartshoe for ambulatory monitoring of physical activity and gait in children with cerebral palsy has been designed [20]. A wide range of algorithms has been used in the literature for detecting stance phases using IMUs. Most of these algorithms use a particular threshold value to detect gait phases. However, the acceleration and angular velocity peaks can vary due to various factors such as age, gender, and physical condition, or even extrinsic factors, such as shoe type or ground surface condition. If the predefined threshold of the IMU is set too low, it will detect all of these peaks as strides. Hence, fixed thresholds are not reliable [21,22,23]. At the same time, proposing a displacement estimation method based on the model of human lower-limb biomechanics combined with sensor data fusion technology, portable wearable lower-limb gait analysis of exercise rehabilitation systems can calculate kinematic parameters and spatio-temporal parameters of the human gait, providing a complete assessment of lower-limb motion and achieving the integration of assessment and rehabilitation training [24,25].

Artificial intelligence technology is also helpful in predicting and diagnosing abnormal body activities. When abnormalities are detected, the generated report is passed to the doctor or specialist. Many researchers have studied the importance of Body Sensor Networks (BSNs) from different perspectives [26,27,28,29,30]. Although research involving wearable MEMS systems has increased significantly in recent years, there has only been a small number of studies attempting to track patients with abnormal gaits using wearable inertial sensors. In addition, due to the difficulties in data acquisition for patients with severe gait abnormalities and related ethical issues, it is difficult to evaluate the efficacy through long-term comparison.

Another problem is that IMUs cannot be worn accurately along the limb direction; therefore, alignment between the IMU coordinate system and the body coordinate system is usually needed to obtain the posture. In addition, to be able to reconstruct human body posture, the posture of various parts of the human body should be unified into a reference coordinate system [31,32]. Therefore, the initial alignment process mainly includes: The alignment between the geographic coordinate system and the reference coordinate system, and the alignment between the IMU coordinate system and the body coordinate system. In this case, previous motion capture methods based on IMU usually require the user to conduct specific alignment movements. However, users such as stroke survivors may not be able to perform certain alignment movements. In order to overcome the shortcomings of existing technology, a gait analysis method without specific alignment motions is expected.

The rest of this paper is organized as follows: Section 3 describes the proposed gait analysis system and methodology. The experimental results are shown in Section 4. Section 5 discusses the potential applications of gait analysis and summarizes this paper.

## 3. Materials and Methods

### 3.1. System Setup

**Figure 3:**
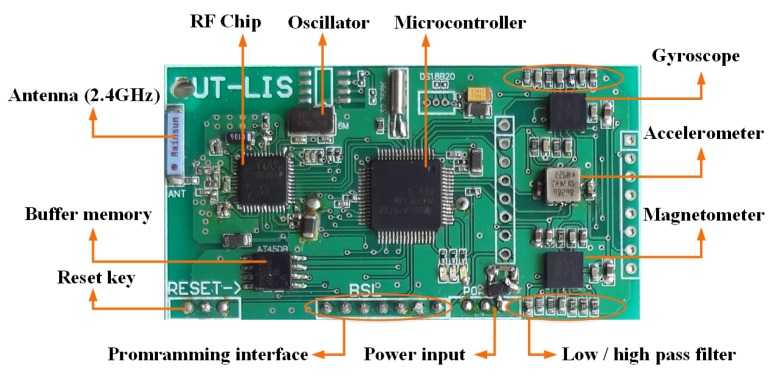
Circuitboard of the proposed sensor node.

Figure 3 shows the gait evaluation sensor module made by our lab. The hardware consists of a low-power microcontroller, inertial sensors, and a 2.4 GHz wireless communication module. The hardware circuit was designed using the above components, and the control procedures were also developed. In order to make this device wearable, system optimization was done to make the system miniature and lightweight. Table 1 summarizes the main sensor parameters.

Inertial data was collected to estimate lower-limb motion. The fourth-order Runge–Kutta method was used to solve the differential equation about the angular velocity and quaternions. The gait phase segmentation was performed automatically. Specifically, the stance phases were first detected by predefined criteria, and then the widely used Zero Velocity Updating (ZUPT) algorithm was adopted to correct errors caused by sensor bias and drift. ZUPT is based on the fact that the foot swings to the stance phase (stationary) periodically during human walking. In this case, the pseudo-measurements of zero velocity were used to calibrate the errors accumulated since the previous ZUPT state [33,34,35]. Next, the rest of the gait phases were easily determined by gyroscope data, which had distinct peak values in every single gait cycle. The footstep of each stride could be correctly detected, thus avoiding significant drift and providing satisfactory position accuracy for different experimental scenarios. The reliability of the proposed system was validated by means of the results obtained from a commercial motion tracking system, namely Optitrack. All data can be saved in Excel or text format for subsequent experimental analysis and data processing.

**Figure 4:**
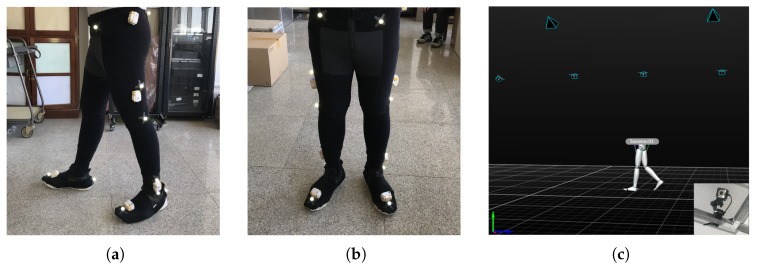
Sensor setup during gait experiments, with optical tracking markers and inertial measurement units (IMUs) attached to the subject; (**a**) Lateral view; (**b**) front view; (**c**) optical motion capture equipment: Software and cameras.

The proposed system is a portable gait analysis system which not only provides convenience of gait analysis to the user, but also guarantees the accuracy of the data results. The applications in physiotherapy, chiropractics, and podiatry are practicable; reliable data can be generated fast enough after patients walk for about 15 m while wearing a pair of IMUs, as shown in Figure 4. The gait assessment only takes several minutes with the wearable sensor module and the software, and the gait report can be obtained immediately. The gait analysis technology is supported by a pattern recognition algorithm which achieves high perceptual accuracy.

### 3.2. Gait Parameter Estimation Using Quaternions

The linear acceleration is used to calculate position by two integral operations in the following formula; note that the gravity component ($g={\left[0,0,9.81\right]}^{T}$ m/s^2^) is properly eliminated from the accelerometer measurements in advance.

In the process of human motion capture, the available information includes the output of each IMU node and the constraints of the human motion characteristics (such as joint rotation degree of freedom constraints, human linkage constraints, etc.).

As for a single sensor node, the state parameter is set as ${\left[q^{T},v^{T},p^{T}\right]}^{T}$, where *q* is a quaternion used to represent the IMU attitude, and *v* and *p* are the velocity and displacement of the IMU, respectively. The corresponding equation of state is as follows:

(1) $$\left\{\begin{array}{c} \dot{q}=\frac{1}{2}q⊗w\\ \dot{v}=q⊗a⊗q^{*}-g^{n}\\ \dot{p}=v, \end{array}\right.$$

where w and a represent the triaxial gyroscope and accelerometer outputs, respectively. $q^{*}$ is the conjugate quaternion, ⊗ indicates quaternion multiplication, and $g^{n}$ indicates gravity vector projection under the ground coordinate.

All lower-limb movements of each step are integrated into one gait cycle in three planes, including the sagittal plane, frontal plane, and transverse plane. Note that the algorithm accuracy of wearable-sensor-based technology is relative to the previous state. However, the inertial data are naturally polluted by various noises, since MEMS inertial sensors are prone to errors. As a result, integral errors accumulate over time. Therefore, real-time correction is necessary. The proposed gait analysis system provides sufficient gait parameters for the user, and it particularly evaluates a person’s gait in the following aspects:

- Cadence
  Cadence is the number of walking steps per minute.
- Stride Length
  The stride length $S_{l}$ is the average distance between the previous touchdown (foot contacts ground) position and the next touchdown position of the same foot, not the distance between the left foot and the right foot.
  (2) $$S_{l}=\frac{\sum_{i=1}^{n}\left(p_{i}^{forward}-p_{i-1}^{forward}\right)}{n},$$
  where $p^{forward}$ represents the computed foot position in the forward direction, while *i* indicates the stride number and *n* is the total stride number.
- Gait Speed
  The gait speed $G_{s}$ is the average walking speed of the foot in the forward direction, and *T* represents the total walking duration.
  (3) $$G_{s}=\frac{p_{n}^{forward}-p_{0}^{forward}}{T}$$
- Max Foot Elevation
  The max foot elevation $E_{m}$ is the maximum height to which the foot is lifted above the ground.
  (4) $$E_{m}=p_{max}^{upward},$$
  where $p_{max}^{upward}$ represents the maximum computed foot position in the upward direction.
- Ankle Range of Motion
  The ankle range of motion represents the angular variation between the shank and instep, which implies the ankle joint control ability.
  (5) $$A_{rom}=\theta^{shank}-\theta^{instep}+\theta_{0}=\int \left(\omega^{shank}-\omega^{instep}\right)dt+\theta_{0}^{shank}-\theta_{0}^{instep},$$
  where $\theta_{0}^{shank}$ and $\theta_{0}^{instep}$ are the initial angles between the lower limbs (shank and instep, respectively) and the gravity direction.
- Stance/Swing Phase Ratio
  Stance and swing phases are the durations in which the foot contacts the ground and leaves the ground. Stance ratio $R_{s}$ is the proportion of stance phase in a gait cycle.
  (6) $$R_{s}=\frac{t_{stance}}{t_{stance}+t_{swing}},$$
  where $t_{stance}$ and $t_{swing}$ represent the duration of the stance phase and swing phase in a gait cycle, respectively.
- Gait Symmetry
  In this study, gait symmetry is explained by $G_{S}$. Gait symmetry shows the coordination of the bilateral movement of the lower limbs. The definition of $G_{S}$ is as follows.
  (7) $$G_{S}=1-\frac{2(X_{a}-X_{s})}{X_{a}+X_{s}},$$
  where $X_{a}$ indicates gait parameters from the affected lower limb and $X_{s}$ represents gait parameters from the sound lower limb. *X* can be cadence, stride length, stance ratio, or any above-mentioned parameter. Unlike other gait parameters, which are calculated via sensor data from unilateral lower-limb movements, the temporal and spatial parameters of bilateral lower-limb movements are included in $G_{s}$. A higher $G_{s}$ score indicates a more symmetrical gait [36,37], while a lower $G_{s}$ score indicates that the gait quality of the patient is still poor and more rehabilitation treatment is needed. Hemiplegia, caused by nervous system diseases such as strokes, is common in clinical practice, causing paralysis of one side of the body and asymmetrical gait. Monitoring gait symmetry is significant in the recovery process, as suggested by rehabilitation physicians.

The integration of acceleration and angular velocity is conducted in a dynamic process, i.e., the swing phase. Note that MEMS accelerometers sense all of the force applied to the sensor, spontaneously including gravity, which is independent of foot movement. In this case, the gravity vector should be derived correctly. During the gait cycle, assuming that the accelerometer offset is invariable, it is feasible to set the foot speed to zero at the end of each swing phase, depending on the periodicity of the person’s walking [38].

The acceleration of foot motion in the swing phase can be denoted as

(8) $$a\left(t\right)=\tilde{a}\left(t\right)+\varepsilon ,$$

where $\tilde{a}$ is the real acceleration and ε is the accelerometer bias error, which is normally assumed as fixed over one gait period. In this case, foot velocity can be set to zero in the stance phase. Then, the three-dimensional foot velocity can be determined by the following formula:

(9) $$v\left(t\right)=\int_{0}^{t}a\left(\tau \right)d\tau =\int_{0}^{t}\left[\tilde{a}\left(\tau \right)+\varepsilon \right]d\tau =\tilde{v}\left(t\right)+\varepsilon t.$$

Acceleration bias error in one single gait cycle can be calculated by

(10) $$\varepsilon =\frac{v\left(T_{sw}\right)}{T_{sw}},$$

where $T_{sw}$ is the duration of the swing phase.

Corrected three-dimensional foot position information can be obtained after velocity correction:

(11) $$p\left(t\right)=\int \tilde{v}\left(t\right)dt.$$

**Figure 5:**
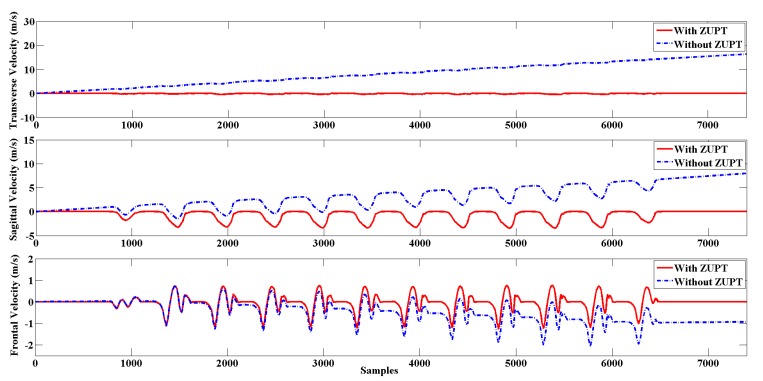
The estimation of three-dimensional foot velocity before applying the Zero Velocity Updating algorithm (ZUPT; blue dotted curve) and after applying the ZUPT (red curve).

Figure 5 shows the effectiveness of the ZUPT algorithm. Note that the ZUPT algorithm effectively eliminates the integral error velocity estimation due to sensor bias. To obtain a smooth motion trajectory, some researchers even introduced smoothers for position estimation [39]; these seem to work well, but a delay would inevitably be introduced, which should be avoided, especially in real-time scenes. In addition, the real effects of smoothers on motion capture research are still disputable [40,41].

### 3.3. Stance Phase Detection via Sensor Fusion

**Figure 6:**
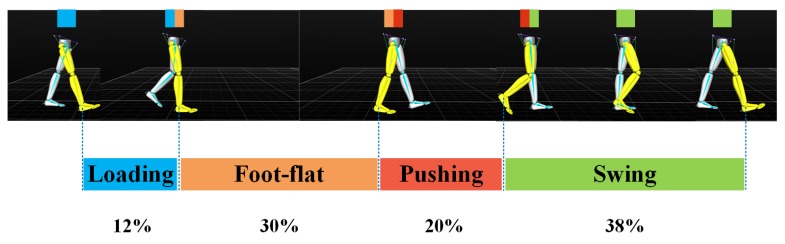
The fundamental temporal parameters: Gait cycle partitioning.

One typical gait cycle is normally divided into a swing phase and stance phase, which can be further divided into three sub-processes, namely loading, foot-flat, and pushing [42,43,44], as shown in Figure 6. The swing phase indicates the period when the foot swings above the ground, and occupies roughly 38% of a single stride time. The stance phase indicates the period of time when at least one foot is contacting the ground, and takes up the remaining 62% of the stride time; double stance phase indicates that the two feet contact the ground at the same time. Note that the double stance phase only exists in walking, and it is not present in running. It was reported that the stance phase and swing phase are about the same length when jogging [45,46].

**Figure 7:**
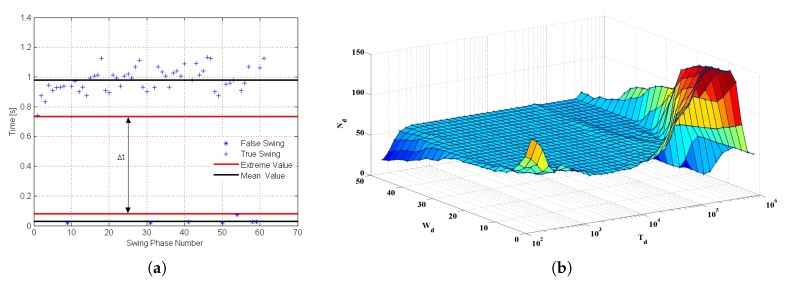
Gait phase detection by the k-mean clustering algorithm. (**a**) Swing phases detection; (**b**) using different window sizes $W_{d}$ and detection thresholds $T_{d}$ to detect step numbers.

**Figure 8:**
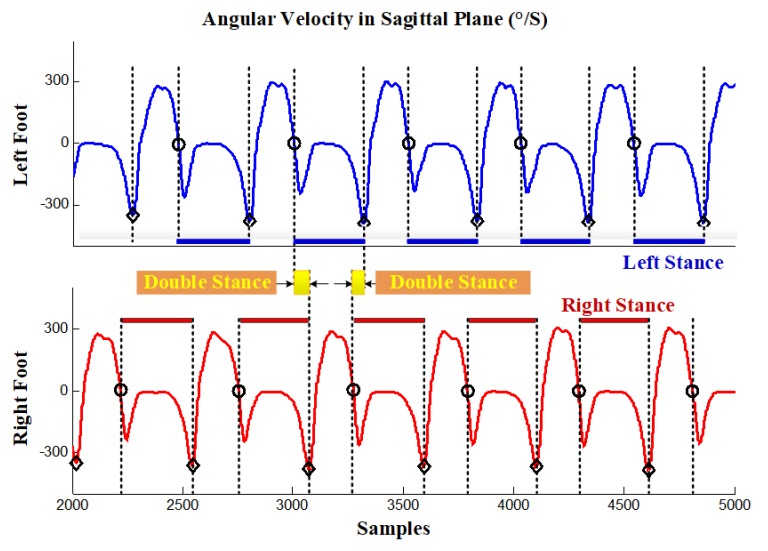
Gait phase detection using angular velocity in the sagittal plane: Single stance phase and double stance phase.

Considering the potential short-term sensor measurement fluctuations, the time constraint is a good complement to validate the attitude phase detection results by evaluating the time duration of each phase. The principle is that a true attitude phase can only be declared if the detection statistics fall within a specified period of time. In general, the fixed time constraint only applies to specific pedestrian data, not to pedestrian data in different walking scenarios. Therefore, an adaptive time constraint is required to provide a robust pedestrian dead reckoning (PDR) solution. In this paper, the k-mean clustering algorithm was used to filter the potential gait phase and obtain the adaptive time constraint parameters automatically. As shown in Figure 7a, the k-mean clustering algorithm effectively divides the detected attitude phase into true and false clustering according to the length of time, thus eliminating the false attitude phase caused by fluctuation. Meanwhile, the extreme value of each cluster is represented by the blue line in Figure 7a. In this case, the k-mean algorithm can generate adaptive time-constrained parameters to eliminate the false attitude phase detection caused by sensor measurement fluctuations. Figure 7b shows the effect of window size *W* on step size detection. A total of 25 different window sizes were selected, and the correct step number is 56. The results show that the optimal window size is neither too large nor too small. Figure 7b shows the effect of the window size $W_{d}$ and detection threshold $T_{d}$ on step number detection; note that the color approaches red when the detected step number exceeds the true value, while the color approaches mazarine when the detected step number is less than the true value. The technical details are described in our previous publication [47,48,49]. Figure 8 shows the gait phase partition resulting from a gait trial performed by a healthy subject.

### 3.4. IMU Installation Location Estimation

For the installation error of IMU, in addition to the installation posture error, there is also the installation position error, which is often ignored due to insufficient information. The installation position of the IMU shows the distance between the IMU and the joint node, which is the premise of establishing the relation between the displacement and the velocity between the joint node and the IMU, and further helps to make full use of the constraints of human movement characteristics and to obtain the motion parameters of the joint.

**Figure 9:**
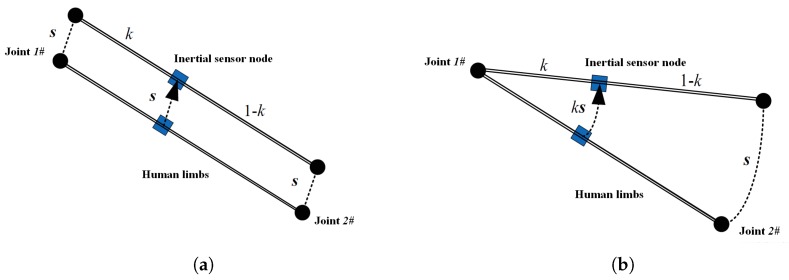
IMU installation position and limb movement types: (**a**) Translational Motion; (**b**) rotational motion.

Normally, the IMU is placed between adjacent human joints; for instance, joints 1 and 2, as shown in Figure 9. Define *k* as the ratio of distance from the IMU to joint 1 and the distance between joint 1 and joint 2, ignoring the tiny displacement between the IMU and body contact site, and the *k* value does not change over time; the *k* value describes the installation position of the IMU. Set the spatial coordinates of adjacent joint 1 and joint 2 as $p_{1}$ and $p_{2}$, and denote the coordinates of the IMU as *p*; then, the following formula is satisfied.

(12) $$p\left(t\right)=p_{1}\left(t\right)+k\left(p_{2}\left(t\right)-p_{1}\left(t\right)\right).$$

Denote *s* as the displacement estimated by inertial navigation, and p(0) as the displacement relative to an arbitrary initial position, i.e., s(t)=p(t)−p(0); then, the state equation of the Kalman filter system is as follows:

(13) $$\left\{\begin{array}{c} \dot{v}=a\\ \dot{s}=v\\ \dot{k}=0 \end{array},\right.$$

where acceleration a is available by IMU measurements. Then, we have the following equation:

(14) $$p\left(0\right)+s\left(t\right)=p_{1}\left(t\right)+k\left(p_{2}\left(t\right)-p_{1}\left(t\right)\right),$$

Finally, the observation equation of the Kalman filter system can be concluded as follows:

(15) $$p_{1}\left(t\right)-p_{1}\left(0\right)=k\left(p_{1}\left(t\right)-p_{1}\left(0\right)-p_{2}\left(t\right)+p_{2}\left(0\right)\right)+s\left(t\right).$$

When human limbs only perform translational motions, the displacement of the IMU is consistent with that of the joint, so it is impossible to determine the installation position of IMU on the limb. In fact, $p_{1}\left(t\right)-p_{1}\left(0\right)-\left(p_{2}\left(t\right)-p_{2}\left(0\right)\right)$ equals zero and *k* is unobservable under the circumstances. In other cases, when the body is rotating, the displacement of the IMU is different from that of the joint, and the proportional relationship between them is defined by *k*. Note that when people move their trunk naturally, it is impossible to perform only translational movements and rotational motion is usually included, especially for the human limbs with higher degrees of freedom; hereby, the observability can be guaranteed. The details of the Kalman filter can be found in the literature [13,50].

By obtaining the installation position of the IMU, the spatial position and velocity relationship between the IMU and adjacent joints can be established, providing important parameters for the subsequent estimation of human posture and joint velocity by using the constraint of human joints and structural constraints.

## 4. Experimental Results

In this paper, we developed a portable sensor system for total lower-limb motion measurement, the calibration methodology, and preliminary data. This sensor is intended for gait analysis and is designed to have a minimal effect on natural gait. A triaxial accelerometer was combined with an array of gyroscopes and magnetometers to form a wearable compliant nine-axis inertial sensor unit. Up eight sensor units can be deployed on each side of the lower limbs. The gait analysis trial participants in this study included 30 healthy adults (22–45 years old) and 30 stroke patients (46–77 years old) from the Second Affiliated Hospital of Dalian Medical University. All subjects were asked to walk continuously on flat ground for more than 15 m in an obstruction-free corridor. The data from the sensors were compared with measurements obtained from a standard optical motion-tracking system. In consideration of the nonlinear effects, the sensor was accurately calibrated with a linear least squares method.

In this pilot study, we originally aimed to collect motion data at different stages to judge the recovery situation. However, it turned out that, at the current stage, it is unpractical to classify all of the cases into different categories by the degree of rehabilitation. Therefore, regarding all of the participated patients, we invited the attending physician to select several cases with significant improvements as the convalescence patient group (CP group). The categorization criteria are based mainly on medical records and clinical observations. Therefore, in Table 2, apart from the healthy participating subjects, 30 patients were further divided into two subgroups, i.e., the stroke survivor (SS) group (24 subjects) and CP group (6 subjects). The corresponding gait parameters between the SS group and CP group show clear differences in terms of mean and extremum values of *s*. Note that a small sample is apparently not statistically significant. To tackle the limitation, we plan to recruit more patients and conduct follow-up gait monitoring, thus eventually investigating the auxiliary diagnosis ability of the proposed system.

Table 2 summarizes the gait posture assessment results of subjects in different groups. Notably, we collected gait data from healthy subjects and patients recovering from strokes. The results showed that the typical gait patterns of stroke patients significantly increased the stance phase and decreased the swing phase, with poor gait symmetry; this is consistent with clinical observations, regardless of the mild gait disorders in mild stroke patients and the severe gait abnormalities in severe stroke patients. These data can be incorporated into electronic medical records and made available to remote clinicians in follow-up clinical studies.

**Figure 10:**
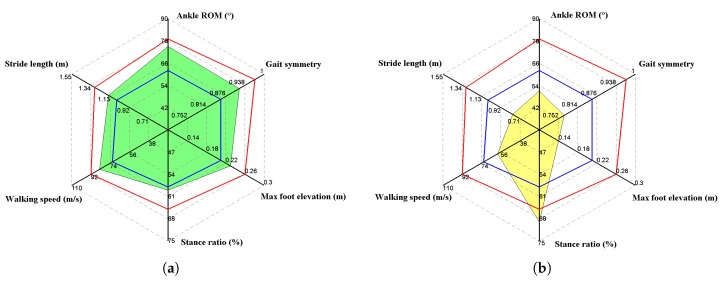
Gait metric comparison results, where red and blue lines represent the minimum and maximum of the corresponding gait parameters: (**a**) Healthy male, age 45 years, weight 76 kg, height 1.74 m, BMI 25.1 kg/m${}^{2}$; (**b**) a stroke survivor, male, age 47 years, weight 69 kg, height 1.72 m, BMI 23.3 kg/m${}^{2}$.

Figure 10 presents the gait metrics of a healthy subject and a typical stroke survivor in terms of radar map, respectively. The two male subjects were in similar age, weight, height, and BMI, thus avoiding the influences of these factors, and the results are comparable. Note that the gait symmetry of the stance ratio is presented. Red and blue lines represent the minimums and maximums of the corresponding gait parameters. Note that the red and blue lines are determined by statistical gait data from a large sample of healthy subjects not only based on the 30 healthy subjects mentioned in the paper, but also from the healthy participants in our previous researches. Red and blue lines in Figure 10 basically determine the normal range. Of course, these lines are not medical evaluation criteria; they are more like visualized references for the clinical staff. Red and blue lines in Figure 10 basically determine the boundary of normal range with regard to different gait parameters. The larger the deviation from normal range, the more serious the gait impairments exist. As for gait analysis, we evaluate gait impairments and walking efficiency of a person with the database by indicating whether the subject’s gait parameters are located within the normal range. It should be noted that in Figure 10, the two male subjects are similar in age, weight, height, and BMI, thus avoiding the influences of these factors, and the results are comparable. In the graphic, the symmetry of the stance phase is presented. Further findings of the comparative experiments are as follows.

Stride length is affected by many factors, including gender, height, age, and physical condition, and it fluctuates over a wide range. In this case, it is not reliable to determine gait abnormality merely according to stride length, though extremely low stride length always indicates gait disorder. The medical staff may refer to extreme values instead of data fluctuations for auxiliary diagnoses. Walking speed is similar to stride length, since it is likewise affected by gender, height, age, and the subject’s intention. Both the stride length and cadence will increase when the subject attempts to increase her/his walking speed. Therefore, stride length and walking speed should be interpreted with reference to each other. For example, a low stride length combined with high walking speed might indicate the attempt to compensate for small steps by intentionally increasing the stride frequency.

With regard to the stance ratio, the different groups show significant diversity. It can be observed that the stance ratios of the control group are very close to the nominal value (60%), and any significant deviation from the reference value indicates gait abnormality. In Figure 10b, the stroke survivor’s stance ratio reaches nearly 70%, which indicates that the subject strove to keep balance by increasing the ratio of the double stance phase.

Ankle range of motion (ROM) reflects the abilities of plantarflexion and dorsiflexion. This factor is widely adopted by orthopedists as an index of diagnosis. Neurological disorders normally weaken ankle control ability, which results in low ankle ROM (reference value around 70${}^{\circ }$). In addition, ankle sprains are frequent injuries that occur among people of all ages. Without proper treatment and rehabilitation, a more severe sprain can weaken the ankle, making it more vulnerable to new injuries and leading to long-term problems. In conclusion, accurate monitoring and follow-up monitoring of ankle ROM are quite helpful in clinical practice.

Another important gait metric is gait symmetry, which is one of the parameters that has separate components for both sides of the lower limbs. The experimental results have shown that there are no remarkable asymmetries for any of the indexes in healthy subjects, with the variability being relatively low, while the stroke survivors always show gait asymmetries to varying degrees. In particular, asymmetries are mostly observed in the max foot elevation and stance ratio indexes. Gait asymmetry may be due to over-adduction of the knee joint, resulting in outward bending of the calf. This way of walking can lead to joint sprains and injuries, and long-term stress imbalance can lead to wear and tear of the outer cartilage of the knee, leading to arthritis. It also puts pressure on the hip joint, leading to chronic pain in the bones while sitting and the formation of so-called x-shaped legs.

**Figure 11:**
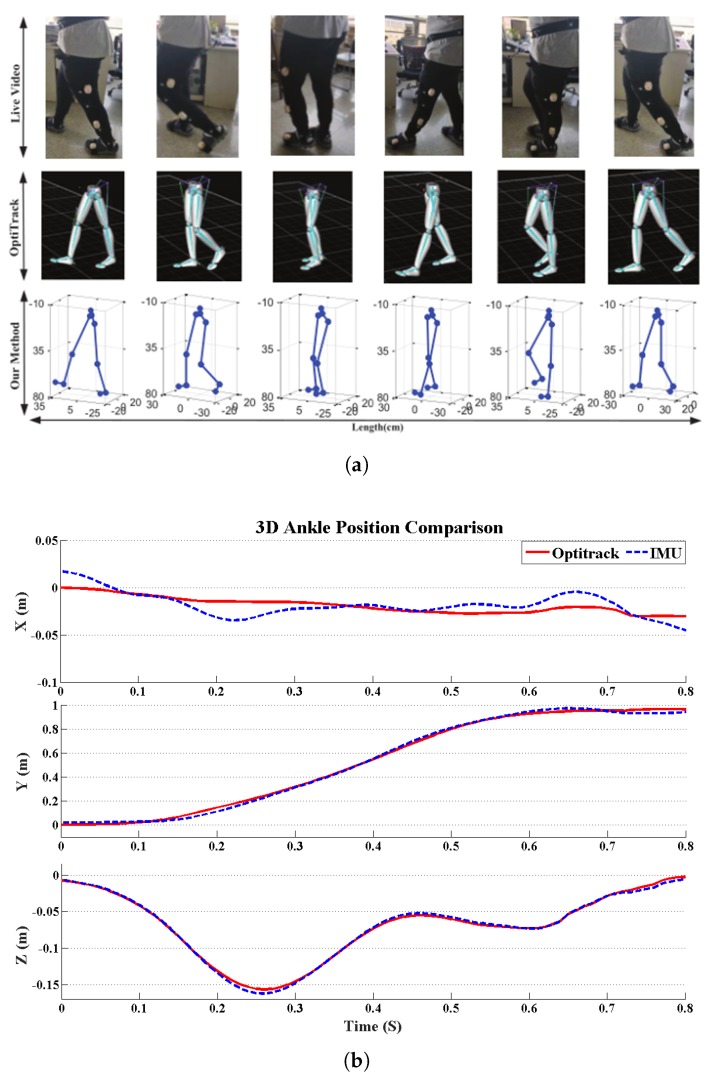
Results of comparative experiments using Optitrack: (**a**) Motion reconstruction; (**b**) ankle position comparison.

The system’s effectiveness was verified by the precision optical motion capture system, namely Optitrack (NaturalPoint, Inc., Corvallis, OR, USA). The accuracy and feasibility of the proposed error correction principle were verified by comparing the experiment with the optical system. Since the ankle is a distal joint of the human lower limbs which can be considered as an inverted pendulum, ankle movements comprehensively reflect the movement of the lower limbs. Hence, ankle position information is normally adopted to reconstruct the walking trajectory in the literature; in this case, the ankle position was compared using the IMU system and the Optitrack system, so as to validate the reliability of the proposed gait analysis system. Figure 11 shows the results of a comparative experiment of the ankle position estimation. A convenience sample of five healthy subjects (three males and two females, age 32.5 ± 6.8 years, weight 67 ± 15 kg, height 1.73 ± 0.21 m, BMI 22.14 ± 4.30 kg/m${}^{2}$) are recruited for the validation experiment. The estimation accuracy of ankle joint position was evaluated in terms of root mean square error (RMSE). The error of the three-dimensional position estimation was less than 0.02 m. Meanwhile, the accuracy of the method in capturing human lower limbs was also verified by the preliminary clinical observations. The experimental results show that the proposed method can be utilized for continuous monitoring of lower-limb motion, including orientation and joint angles.

## 5. Discussions and Conclusions

Our preliminary clinical trial findings highlight the importance of a wearable inertial BSN as a useful tool for assessing gait ability in stroke patients with varying degrees of gait impairment. The paper mainly proposes a measurement method that is capable of quantifying the spatial and temporal parameters of gait, and the gait assessment results are in accordance with clinical observations and optical device outputs.

IMU data calibration is an essential part of gait extraction feature estimation. Note that the accuracy of IMUs has been improved; measurement errors are unavoidable with current technology especially when using MEMS sensors. We detected the zero-velocity, a domain-specific assumption in the non-stationary period of the stance phase, to reduce the integration drift. Other possible areas of error can arise from friction from the relative movement of the sensors against clothing or shoes. We have taken measures to reduce errors as much as possible by fitting sensors tightly with hook-and-loop fasteners, and we used adaptive step detection techniques to bring out the gait phases, as shown in Figure 7. In addition, via modeling of human biomechanical models, the motion of lower limbs in 3D space was reconstructed. Our research validating the IMU against a commercial optical device, Optitrack, the gold standard of human motion capture, reported a very close comparison in accuracy for measuring the orientation and position. The step-size position estimation accuracy was validated. Simultaneously, the 3D human motion reconstruction by our IMU-Meanwhile, smartphone can be used based method is in good agreement with the model reconstructed by Optitrack, as shown in Figure 11a, which indicates that our method can track gait motion with reasonable accuracy.

**Figure 12:**
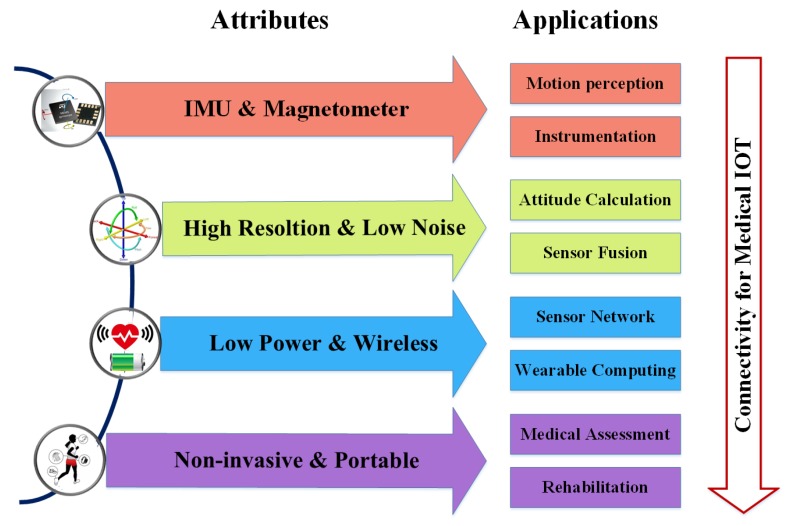
Medical Internet of Things and gait analysis.

In literature, the most concerned gait parameters are stance ratio, foot elevation, and ankle ROM, as suggested by rehabilitation physicians [51,52]. Abnormal gait often appears as a prolongation of stance phase ratio to increase walking stability. For the elderly and stroke patients, as the body flexibility and balance decrease, they unconsciously minimize the time it takes lower limbs to lift off the ground so as to avoid falling. So, it is very common that the elderly and patients tend to perform a rubbing gait, which appears as significantly reduced foot elevation and ankle ROM, as shown in Figure 10b. Nevertheless, it is not reliable to determine gait abnormality merely on gait parameters of unilateral lower-limb movements; therefore, gait symmetry that reflects the specific gait parameters of bilateral lower-limb movements are widely recognized as more convincing indicators of disease progression. To sum up, gait analysis offers an effective tool for health management and rehabilitation assessment. In fact, our body motion tells everything; as part of the data input in the field of rehabilitation, the development of motion capture technology has made it much easier for clinical applications to digitally record people’s movements for analysis, training, and guidance. In fact, many scholars have used Wii, Kinect, PS2, and other motion sensing devices to develop rehabilitation projects, such as limb rehabilitation, balance training, and cognitive tests. Obviously, it is impossible for patients to carry active Kinects and other devices. Motion capture technology based on MEMS sensors solves the portability problem of motion data acquisition. Medical Internet of Things applications have huge potential, as shown in Figure 12.

This is merely a pilot study and the numbers of subjects are limited; many more participants are needed in further research. Moreover, motor disorders due to stroke require a long-term rehabilitation course. The time span of this experiment was relatively short, and the improvement of symptoms was not obvious for some subjects. Further research will focus on investigating its efficacy as an early prediction of central nervous diseases or peripheral neuropathy. In addition, we plan to develop interesting visual rehabilitation training games and customizable extended rehabilitation items. As an input tool for virtual reality, patients can immerse themselves in motion sensing games and conduct rehabilitation training at the same time. Patients no longer have to endure boring recovery actions, and their compliance can be improved accordingly. It is widely recognized that wearable sensor-based technology will have profound impacts on our daily lives, especially because nearly every country deals with aging populations and the rehabilitation of the elderly. Companies including Google and Nike have developed a variety of commercial wearable smart devices. However, few commercial off-the-shelf devices are used for medical purposes. Therefore, developing medical wearable devices for dynamic estimation of human activity is a promising field.

## Figures and Tables

**Table 1:** Sensor performance specifications.

Unit	Accelerometer	Gyroscope	Magnetometer
Dimensions	3 axes	3 axes	3 axes
Dynamic Range	±50 m/s${}^{2}$	±1200 ${}^{\circ }/s$	±750μT
Bandwidth (Hz)	30	40	10
Linearity (% of FS)	0.2	0.1	0.2
Bias stability (unit 1σ)	0.02	1	0.1

**Table 2:** Gait posture assessment results of healthy subjects (HS), stroke survivors (SS), and convalescence patients (CP).

Gait Parameters	HS	SS	CP
Ankle range of motion (ROM) (${}^{\circ }$)	72±10	52±18	59±16
Stride length (m)	1.19±0.13	0.87±0.31	1.01±0.24
Cadence (steps/min)	112±11	85±24	97±19
Walking speed (m/s)	1.34±0.175	0.91±0.28	1.16±0.22
Stance ratio (%)	59±7	73±21	65±14
Max foot elevation (m)	0.24±0.06	0.13±0.12	0.15±0.09
Gait symmetry (stance)	0.93±0.06	0.61±0.13	0.78±0.15

## Abbreviations

The following abbreviations are used in this manuscript:

MEMS	Micro-Electro-Mechanical Sensor
BSN	Body Sensor Network
IMU	Inertial Measurement Unit
ZUPT	Zero Velocity Updating
